# Telemedicine diagnosis of acute respiratory tract infection patients is not inferior to face-to-face consultation: a randomized trial

**DOI:** 10.31744/einstein_journal/2022AO6800

**Published:** 2022-05-18

**Authors:** Tarso Augusto Duenhas Accorsi, Flavio Tocci Moreira, Carlos Henrique Sartorato Pedrotti, Karine De Amicis, Renata Farias Vidigal Correia, Renata Albaladejo Morbeck, Fernanda Ferreira Medeiros, José Leão de Souza, Eduardo Cordioli

**Affiliations:** 1 Hospital Israelita Albert Einstein São Paulo SP Brazil Hospital Israelita Albert Einstein, São Paulo, SP, Brazil.

**Keywords:** Telemedicine, Respiratory tract infections, Emergency medical services, Referral and consultation, COVID-19, Coronavirus infections

## Abstract

**Objective:**

To analyze telemedicine diagnostic accuracy in patients with respiratory infections during COVID-19 pandemic compared to face-to-face evaluation in the emergency department.

**Methods:**

Randomized, unicentric study between September 2020 and November 2020 in patients with any respiratory symptom (exclusion criteria: age >65 years, chronic heart or lung diseases, immunosuppressed). Patients were randomized 1:1 for brief telemedicine followed by face-to-face consultation or direct face-to-face evaluation. The primary endpoint was the International Classification of Diseases code. The secondary analysis comprised length of stay, diagnostic test ordering, medical prescription, and proposed destination.

**Results:**

Ninety-eight patients were enrolled. The mean age was 36.3±9.7 years old, 57.1% were women, and 81.6% had diagnostic test ordered. Mean grouped by International Classification of Diseases code for upper respiratory tract infection, pharyngotonsillitis, and sinusitis showed no difference between study groups or secondary endpoints. The Telemedicine Group was representative of the population usually evaluated in this center. In the Telemedicine Group (n=48), 18.7% patients would be referred for evaluation at the emergency department. The distribution of diagnoses by telemedicine was 67.4% for upper respiratory tract infection, 2.3% for pharyngotonsillitis, and 0% for sinusitis, being statistically similar to the subsequent face-to-face assessment, respectively: 72.1%, 11.6% and 7% (Kappa 0.386 [95%CI: 0.112-0.66]; p=0.536). Telemedicine ordered COVID-19 molecular (RT-PCR) tests in 76.5% *versus* 79.4% in face-to-face evaluation (Kappa 0.715 [95%CI: 0.413-1]; p>0.999).

**Conclusion:**

Diagnostic telemedicine consultation of low-risk patients with acute respiratory symptoms is not inferior to face-to-face evaluation at emergency department. Telemedicine is to be reinforced in the health care system as a strategy for the initial assessment of acute patients. **ClinicalTrials.gov Identifier:** NCT04806477

## INTRODUCTION

Telemedicine (TM) has become a fundamental resource in the health system due to its cost-effective and easy way of providing a medical evaluation to the population through immediate actions.^( 1 )^ Observational studies have shown that TM provides good accuracy in the clinical assessment of several conditions.^( 2 )^ However, randomized controlled trials with TM lack support of a high level of evidence.^( 3 )^

Respiratory tract infection (RTI) symptoms are common, and most are a consequence of low-risk upper respiratory tract viral infections, comprising a significant percentage of face-to-face medical care at the emergency department (ED).^( 4 )^ Emergency services are overcrowded, usually due to high volume of low-acuity presentations and limited access to primary care; this relates to worsening the prognosis of all emergencies.^( 5 , 6 )^ Strategies to minimize occupancy in the ED, especially prehospital care, should be implemented.

One of these strategies is TM, which demonstrates incredible potential in this setting.^( 7 )^ Regarding patients with suspected RTI, TM can help oligosymptomatic patients follow the correct guidelines at home and improve adequate referral.^( 8 , 9 )^Nonetheless, direct-to-consumer TM diagnostic accuracy of RTI is still not well studied and time spent on evaluation, test ordering, and general guidance provided.

During the coronavirus disease (COVID-19) pandemic, TM adoption as an initial assessment of patients was emphasized to support government quarantine measures, reduce contagion and costs, and highlight the importance of confirming the diagnostic accuracy strategy.^( 10 )^

Due to the general low accuracy of physical examination in low-risk RTI patients, most of the diagnostic rationale originates from the patient’s history. Thus, this study hypothesizes that direct-to-consumer TM consultation diagnostic accuracy is not inferior to face-to-face evaluation at the ED.^( 11 )^

## OBJECTIVE

This randomized study aims to compare telemedicine diagnosis with face-to-face evaluation in patients with suspected respiratory tract infection who spontaneously sought evaluation at an emergency department.

## METHODS

### Trial oversight

This unicentric trial had a randomized, non-inferiority, open-label design with blinded adjudication of the primary outcome. The ED study was performed at *Hospital Israelita Albert Einstein* (HIAE) in São Paulo, (SP), Brazil, which is a private general hospital, unreferenced, with a predominance of young, low-complexity patients, where general emergency physicians perform a medical evaluation.

The TM center is located at the same institution, functioning as a virtual ED with independent routines and professionals. Both services adopt the Cerner Millennium electronic medical records platform, stewardship protocols, and high adherence to international guidelines.

Data were gathered and confidentially stored by TM physicians unrelated to the local face-to-face care team. The first, second, third, and last authors who wrote the manuscript initial draft had full access to all data and reviewed the manuscript. All analyses were performed by the TM center coordinating the trial. All authors decided to submit the manuscript for publication and attest to the data integrity and accuracy and the trial fidelity to the protocol. No one who is not an author contributed to the writing of the manuscript.

The trial protocol was approved by the institutional ethics board with registration # CAAE: 34172720.4.0000.0071, protocol 4.161.528 and named TeleIVAS (IVAS is the acronym for RTI, in Brazilian Portuguese), and all data can be accessed in the institutional digital records. The study was also registered at ClinicalTrials.gov identifier: NCT04806477 and has no funders. There are no changes to methods or outcomes after trial commencement.

### Patients

This study included adults (≥18 years old) who had at least one acute symptom compatible with RTI (sore throat, nasal obstruction, rhinorrhea, new or recent-onset cough, sputum, hoarseness, dyspnea) with or without systemic symptoms (fever ≥38^o^C, chills, sweating, myalgia) that motivated spontaneous face-to-face evaluation at the ED. Eligible patients had all procedures and data collected during the ED stay.

The main exclusion criteria were age >65 years old, chronic respiratory disease diagnosis (chronic obstructive pulmonary disease, asthma, and interstitial lung disease), previous congestive heart failure diagnosis, HIV/AIDS, active cancer, type I *diabetes mellitus* , any immunosuppressant use, chronic cough and emergency room referral after nursing triage. The study sought low-risk patients screening, representing a large part of the ED arrivals and those in whom TM is most indicated. All patients provided written informed consent.

### Trial procedures

From September to November 2020, eligible patients were randomly assigned to receive a brief TM consultation followed by face-to-face evaluation (TM-ED Group) or standard face-to-face evaluation (ED Group) in a 1:1 ratio in an app-based automated randomization system (Randomizer 1.2, Darshan Institute of Engineering & Technology, Gujarat, India). The app provided the random allocation sequence immediately after inclusion in the study. Based on institutional protocols and clinical judgment, face-to-face medical assessment, with all necessary resources, was considered gold standard and was performed in all patients.

Patients assigned to receive TM first (TM-ED Group) were evaluated in the triage room immediately after nursing initial protocols and enrolled using a digital tablet connected to an institutional wireless network (Apple iPad, California, CA, USA). The patient waited in a virtual waiting room, and the evaluation was performed by the TM center team, according to TM center protocols, using a licensed HIPAA-compliant platform (InTouch Health, California, CA, USA). The evaluation was recorded in the institutional electronic records but not made readily available to the local ED medical team, who remained blind to the test randomization.

The TM consultation was interrupted when the doctor made the diagnostic hypothesis, which was not informed to the patient. This arrangement aimed at partial blinding and to reduce bias in sequential face-to-face care. The remote consultation data were tabulated through specific checkboxes for respiratory complaints and free text according to clinical judgment, while orientations, drug prescription suggestions, and possible referrals were covered in distinct form fields.

Institutional protocol for remote evaluation of RTI and COVID-19 could be easily consulted in an electronic tab during the consultation. A decision support system was also available, integrated into the electronic system according to the diagnostic hypothesis. Immediately after TM consultation, the patient was always taken to the usual face-to-face care flow at the ED. No patients were exclusively discharged after TM.

All study participants were assigned to receive standard face-to-face evaluation according to the institutional care flow in ED without any contact with the TM center. Despite the same patient in the TM-ED Group also being evaluated in person, allowing comparison, a Control Group (ED Group) with standard face-to-face service was established, minimizing systematic errors.

The ED staff had no prior orientation, and only the screening nurse knew about the protocol, and all patients in both study arms had their final diagnosis based on face-to-face care at the ED. The only way for the ED physician to know about the protocol was to be informed by the patient himself, which could not be measured.

All institutional ED or TM assessments involve filling out the final diagnosis using the International Classification of Diseases (ICD-10) code before discharge or admission. Length of stay (defined from the beginning of the nurse triage until the ED discharge), tests ordered, discharge instructions, and the medical prescription were compiled in the electronic medical records.

Patient participation ended with ED discharge, with no follow-up. Telemedicine also did not check tests. The study aimed to evaluate the hypothesis generation and initial management. The study ended after achieving the previously estimated sample size.

### Primary and secondary outcomes

The primary outcome was the final evaluation ICD-10 code diagnosis assessed by the electronic medical record. For the aggregation of most prevalent RTI with similar pathophysiologic characteristics, three diagnostic groups were defined based on ICD-10 codes: RTI, including COVID-19 (B34.2, B34.9, B97.2, J00, J04, J06, J11, J20, J30, J39, U07.1); Acute Pharyngotonsillitis (J02-J03.9) and Acute Sinusitis (J01-J01.9). The secondary analysis comprised length of stay and proposed test ordering, drug prescription, and referral.

### Statistical analysis

Diagnostic accuracy of TM assessment was defined by the proportion of patients with concordant diagnoses obtained by TM and face-to-face assessment. Non-inferiority of the diagnostic accuracy was investigated by proportion test (H0: P ≤ 70% *versus* H1: P > 70%), considering an expected accuracy of 90% and a non-inferiority margin of 20%. A previous study observed high concordances between TM and in-situ diagnosis for a clinical condition.^( 12 )^ This study estimated a 90%, diagnostic accuracy of RTI by TM, based on the low proportion of critically ill patients with acute respiratory complaints, high diagnostic capacity by anamnesis for virus infection, and the observed low referral rate by TM in the service.^( 9 , 13 )^ Sample size was obtained to demonstrate the non-inferiority of the TM diagnosis accuracy with a clinically acceptable variation of up to 20% less in the estimated accuracy (non-inferiority margin of 20%). The enrollment of 37 patients per group would provide approximately 85% chance to detect a non-inferiority margin in the primary analysis, with a unilateral significance level of 2.5%. The IBM-SPSS for Windows version 22.0 software was used for statistical calculations.

Continuous variables were expressed as means and standard deviation, and categorical variables were presented as counts and percentages. There was no missing data. The demographic analysis was performed by χ^2^ test and *t-* Student test. International Classification of Diseases codes, tests ordered, and the prescription between groups was compared through χ^2^ test, Fisher’s exact test, likelihood ratio test, and Mann-Whitney test. McNemar test was used to compare the distribution of diagnoses, test ordering, and drug prescription between teleconsultation and subsequent face-to-face evaluation. Cohen’s Kappa coefficient was used to measure inter-rater reliability for ICD-10 codes between groups. Values with p<0.05 were considered statistically significant, and the established confidence interval was 95%.

## RESULTS

### Patients

**Figure 1:**
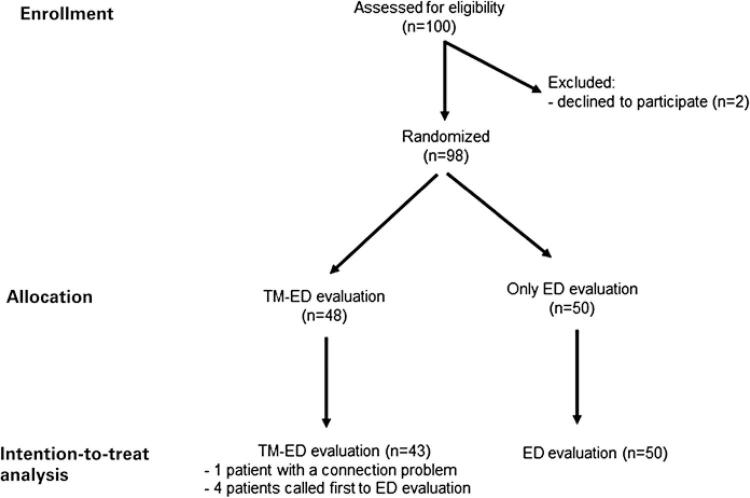
Participants flow diagram

ED: emergency department; TM: Telemedicine.

From September 14, 2020, to November 20, 2020, 100 patients were enrolled and randomly assigned to receive TM consultation and blinded to subsequent face-to-face evaluation (TM-ED Group) or direct face-to-face evaluation (ED Group). Two patients withdrew consent. Four patients in the TM-ED Group were called for face-to-face evaluation before TM, and one had connection problems. These patients were analyzed on an intention-to-treat basis ( Figure 1 ).

The two groups were similar concerning median age (36.3±9.7 years) and gender (57.1% female). Patients were predominantly young and without comorbidities. Of the trial patients, the ED evaluation ordered at least one diagnostic test in 80 patients (81.6%). About 73.5% of all tests were reverse transcription polymerase chain reaction (RT-PCR) for COVID-19, 22.4% for complete blood count, 20.4% C-reactive protein, 16.3% point-of-care *Streptococcus* test, 13.3% D-dimer, 11.2% creatinine, 11.2% chest radiography, 11.2% chest computed tomography, 7.1% oropharyngeal cultures, 5.1% COVID-19 serology, 5.1% influenza rapid test, and 9.2% other blood tests. The grouped ICD-10 distribution of ED diagnostics was 70.4% for upper airway infection, 15.3% for pharyngotonsillitis, and 4.1% for sinusitis. Only 10.2% of patients received other diagnostics codes ( Table 1 ).

**Table 1:** Patient demographics, final diagnosis, tests ordered, emergency department length of stay, and prescription

Variable	Study groups		Total (n=98)	p value
	TM-ED (n=48)	Only ED (n=50)		
Age (years)				0.383*
mean ± SD	35.4±9.9	37.2±9.5	36.3±9.7	
Gender				0.145^☨^
Female	31 (64.6)	25 (50)	56 (57.1)	
Male	17 (35.4)	25 (50)	42 (42.9)	
Final ICD-10 ED diagnosis				0.658^#^
Upper airway infection	33 (68.8)	36 (72)	69 (70.4)	
Pharyngotonsillitis	8 (16.7)	7 (14)	15 (15.3)	
Sinusitis	3 (6.3)	1 (2)	4 (4.1)	
Others	4 (8.3)	6 (12)	10 (10.2)	
Chest CT scan				0.374^☨^
No	44 (91.7)	43 (86)	87 (88.8)	
Yes	4 (8.3)	7 (14)	11 (11.2)	
Chest radiography				0.695^☨^
No	42 (87.5)	45 (90)	87 (88.8)	
Yes	6 (12.5)	5 (10)	11 (11.2)	
COVID-19 RT-PCR				0.300^☨^
No	15 (31.3)	11 (22)	26 (26.5)	
Yes	33 (68.8)	39 (78)	72 (73.5)	
*Streptococcus * test				0.929^☨^
No	40 (83.3)	42 (84)	82 (83.7)	
Yes	8 (16.7)	8 (16)	16 (16.3)	
Oropharyngeal culture				0.712^†^
No	44 (91.7)	47 (94)	91 (92.9)	
Yes	4 (8.3)	3 (6)	7 (7.1)	
Hemogram				0.913^☨^
No	37 (77.1)	39 (78)	76 (77.6)	
Yes	11 (22.9)	11 (22)	22 (22.4)	
C-reactive protein				0.918^☨^
No	38 (79.2)	40 (80)	78 (79.6)	
Yes	10 (20.8)	10 (20)	20 (20.4)	
D-dimer				0.415^☨^
No	43 (89.6)	42 (84)	85 (86.7)	
Yes	5 (10.4)	8 (16)	13 (13.3)	
Creatinine				0.804^☨^
No	43 (89.6)	44 (88)	87 (88.8)	
Yes	5 (10.4)	6 (12)	11 (11.2)	
COVID-19 serology				>0.999^†^
No	46 (95.8)	47 (94)	93 (94.9)	
Yes	2 (4.2)	3 (6)	5 (5.1)	
Influenza test				>0.999^†^
No	46 (95.8)	47 (94)	93 (94.9)	
Yes	2 (4.2)	3 (6)	5 (5.1)	
Other serum tests				0.738^†^
No	43 (89.6)	46 (92)	89 (90.8)	
Yes	5 (10.4)	4 (8)	9 (9.2)	
Admission				0.490^†^
No	47 (98)	50 (100)	97 (99)	
Yes	1 (2)	0 (0)	1 (1)	
Antibiotic prescription				0.427^☨^
No	37 (77.1)	35 (70)	72 (73.5)	
Yes	11 (22.9)	15 (30)	26 (26.5)	
Corticosteroids prescription				0.546^☨^
No	37 (77.1)	41 (82)	78 (79.6)	
Yes	11 (22.9)	9 (18)	20 (20.4)	
Antibiotic class				0.742^#^
Penicillin/Amoxicillin	5 (45.5)	7 (46.7)	12 (46.2)	
Cephalosporin	3 (27.3)	4 (26.7)	7 (26.9)	
Azithromycin	3 (27.3)	3 (20)	6 (23.1)	
Other	0 (0)	1 (6.7)	1 (3.8)	
ED length of stay (minutes)				
mean±SD	80.8±89	97.9±88.1		0.167^£^

* *t* student test; ^☨^ χ^2^ test; ^#^ Likelihood ratio test; ^†^ Fisher’s exact test; ^£^ Test Mann-Whitney. ED: emergency department; TM: Telemedicine; SD: standard deviation; CT: computed tomography; ICD-10: International Classification of Diseases-code; RT-PCR: reverse transcription polymerase chain reaction; COVID-19: coronavirus disease.

Regarding prescription, 26.5% of patients received antibiotics and 20.4% oral corticosteroids. Emergency department length of stay was 89.5±88.5 minutes. Only one patient was admitted. There was no statistical difference between the two groups concerning these tests, ED length of stay, final ED grouped ICD-10 diagnosis, and prescription ( Table 1 ). This comparison aimed to demonstrate that the TM-ED Group had a similar patient profile and regular care practice to the ED Group, representing our institution’s usual population.

### Trial evaluation

#### Primary outcome

Diagnostic distribution by Telemedicine in TM-ED Group was 29 (67.4%) for upper airway infection, 1 (2.3%) for pharyngotonsillitis and 0 (0%) for sinusitis and was statistically similar to the subsequent face-to-face assessment, respectively, 31 (72.1%), 5 (11.6%) and 3 (7%); Kappa was 0.386 [95% confidence interval -95%CI: 0.112-0.66]; p=0.536 ( Table 2 ).

**Table 2:** Diagnostic correlation between Telemedicine and face-to-face visits in TM-ED Group

ED diagnoses	TM diagnoses						Kappa 95%CI
	UAI	Pharyngotonsillitis	Others		Total	p value	
UAI	29 (67.4)	1 (2.3)	1 (2.3)	31 (72.1)		0.536^¥^	0.386
Pharyngotonsillitis	1 (2.3)	4 (9.3)	0 (0)	5 (11.6)			(0.112-0.66)
Sinusitis	2 (4.7)	0 (0)	1 (2.3)	3 (7)			
Others	4 (9.3)	0 (0)	0 (0)	4 (9.3)			
Total	36 (83.7)	5 (11.6)	2 (4.7)	43 (100)			

^¥^ McNemar test.

ED: emergency department; TM: Telemedicine; UAI: upper airway infection; 95%CI: confidence interval.

#### Secondary outcomes

Coronavirus disease 19 RT-PCR was ordered by TM in 26 (76.5%) *versus* 27 (79.4%) in face-to-face evaluation, Kappa 0.715 [95%CI: 0.413-1]; p>0.999 ( Table 3 ). Telemedicine trended towards less antibiotic prescription: 2 (5.9%) *versus* 6 (17.6%), Kappa 0.452 [95%CI: 0.031-0.873]; p=0.125 ( Table 4 ). Additional information regarding corticosteroids prescription, ED length of stay and proposed destination is available in table 1 .

**Table 3:** Correlation of COVID-19 reverse transcription polymerase chain reaction ordered by Telemedicine and face-to-face at emergency department in TM-ED Group

ED RT-PCR request	TM RT-PCR request		Total	p value	Kappa 95%CI
	No	Yes			
No	5 (14.7)	2 (5.9)	7 (20.6)	>0.999^¥^	0.715
Yes	1 (2.9)	26 (76.5)	27 (79.4)		(0.413-1.0)
Total	6 (17.6)	28 (82.4)	34 (100)		

^¥ ^McNemar test.

ED: emergency department; TM: Telemedicine; UAI: upper airway infection; 95%CI: 95% confidence interval RT-PCR: reverse transcription polymerase chain reaction.

**Table 4:** Correlation of antibiotics prescribed by Telemedicine and face-to-face at emergency department in TM-ED Group

ED antibiotic prescription	TM antibiotic prescription		Total	p value	Kappa 95%CI
	No	Yes			
No	28 (82.4)	0 (0)	28 (82.4)	0.125^¥^	0.452
Yes	4 (11.8)	2 (5.9)	6 (17.6)		(0.031-0.873)
Total	32 (94.1)	2 (5.9)	34 (100)		

^¥^ McNemar test.

ED: emergency department; TM: Telemedicine; 95%CI: confidence interval.

## DISCUSSION

This was the first study to date to randomly analyze the diagnostic accuracy of TM assessment of suspected RTI. A common reason for ED visits was chosen, boosted by pandemic, in a center with a predominance of low-risk subjects, where there is more significant potential for TM.^( 14 )^ Regarding patients with suspected RTI during the COVID-19 pandemic, the TeleRTI trial demonstrated that TM diagnostic evaluation is not inferior to face-to-face evaluation at ED. No clinical adverse events were reported associated with this study.

In this study involving patients with acute respiratory symptoms who had sought ED, those who received TM evaluation were markedly more quickly evaluated (median of 5 minutes and 30 seconds of remote assistance). No grouped ICD-10 statistically significant diagnostic differences were found. However, the Kappa correlation coefficient was poor. Grouped ICD-10 was selected for the primary outcome because it usually represents the final integrated clinical judgment and strict medical prescription correlation.^( 15 )^

Compilation analyses of patients referred symptoms and descriptive physical examination may not be related to the final diagnosis.^( 16 )^ Non-ideal final RTI diagnostic correlation can be explained by the diversity of clinical manifestations of viral respiratory tract infections, and regardless of the ICD-10 received, prognosis is generally good, and symptomatic treatment is the standard strategy.^( 17 , 18 )^ With the single exception of physical examination, the quality of care in TM should be the same as in-person care.^( 19 )^ Even when possible, when there are no red flags on anamnesis, physical examination of suspected RTI (including COVID-19) adds low value to the final diagnosis and influences the antibiotics prescription very little.^( 11 )^ Thus, most ICD-10 diagnoses for low-acuity RTI have the same prognosis and suggested guidelines prescription. It was demonstrated in this study when patients had the same destination and prescription strategy regardless of medical evaluation strategy.

Another critical study observation is that a quick TM evaluation without other diagnostic tests was sufficient to define most patients’ diagnoses and provide appropriate treatment. It is supported by previous studies showing that additional laboratory or imaging tests, ordered without clinical suspicion, rarely add value in the diagnosis of bacterial infection or prediction of a poor prognosis.^( 20 , 21 )^ The only test ordered by TM was COVID-19 RT-PCR, which had a very high correlation with the same test-ordering rate by face-to-face ED evaluation (Kappa=0.715). This ordering routine was intended to guide patients to proceed with the test realization in a laboratory of choice and keep following general recommendations at home. Emergency department evaluation had the same proportion of COVID-19 RT-PCR tests ordered, but the result also was not available during ED stay, making no apparent benefit of in-situ tests for diagnosis hypothesis formulation. Most of the other tests ordered at ED were for patient risk stratification.

Unfortunately, ED physicians usually face many conditions that may imply poor guideline adherence, inadequate test ordering, and inappropriate prescribing.^( 22 )^ In this study, ED imaging strategy, with either chest X-ray or tomography scan and lab tests, did not rearrange patient risk, and 98% of participants were discharged with no significant difference from the suggested TM evaluation. Only one patient was admitted for investigation of possible tonsillitis abscess, whose TM evaluation indicated antibiotic treatment. This finding reinforces the low accuracy of commonly ordered additional tests at ED when the clinical evaluation pre-test probability is low for a bacterial infection or respiratory distress.^( 23 )^

It has recently been shown that direct-to-consumer TM encounters are associated with high adherence to best practice guidelines, and a low antibiotic prescription rate in low-risk patients.^( 24 )^ So, remote assessment following strict quality protocols may avoid unnecessary test requests and manage patients without red flags well.

Another significant TM advantage in low-risk patients suspected of RTI is reduction of exposure to contagion at ED and the need for personal protective equipment.^( 25 , 26 )^

This study has several limitations: there was no follow-up after the first encounter and no review of pending laboratory tests or image testing. Although patient management can be considered statistically equivalent at an individual level, specific issues leading to safer measures in face-to-face visits might not have been measured in the studied population. Additionally, this was a single-center study performed in a highly controlled environment, and there should be caution in generalizing the results of this study.

The exponential increase in TM consultations due to the pandemic was not accompanied by equivalent scientific production to support this medical practice mode.^( 27 , 28 )^ Telemedicine provides quick and easy access to medical care, and this study supports that a protocol-based remote assessment allows good diagnostic accuracy and safety in typical patients’ complaints. Telemedicine has transitioned to a multimodal health system due to rapid technological advances, offering greater possibilities and convenience for patients and medical staff. The present results may provide a valuable reference for other TM centers. In suspected RTI in low-risk patients during the pandemic, TM assessment allows diagnostic accuracy comparable to face-to-face evaluation.

## CONCLUSION

Diagnostic telemedicine consultation of low-risk patients with acute respiratory symptoms is not inferior to face-to-face evaluation in the emergency department. Telemedicine must be reinforced in the health care system as a cost-effective strategy for the initial assessment of acute patients.

## References

[1] .Hollander JE, Carr BG. Virtually perfect? Telemedicine for Covid-19. N Engl J Med. 2020;382(18):1679-81. 10.1056/NEJMp2003539 32160451

[2] .Hersh W, Helfand M, Wallace J, Kraemer D, Patterson P, Shapiro S, et al. A systematic review of the efficacy of telemedicine for making diagnostic and management decisions. J Telemed Telecare. 2002;8(4):197-209. Review. 10.1258/135763302320272167 12217102

[3] .Ekeland AG, Bowes A, Flottorp S. Effectiveness of telemedicine: a systematic review of reviews. Int J Med Inform. 2010;79(11):736-71. Review. 10.1016/j.ijmedinf.2010.08.006 20884286

[4] .Irwin RS, French CL, Chang AB, Altman KW; CHEST Expert Cough Panel*. Classification of cough as a symptom in adults and management algorithms: CHEST Guideline and Expert Panel Report. Chest. 2018;153(1):196-209. 10.1016/j.chest.2017.10.016 PMC6689094 29080708

[5] .Bond K, Ospina MB, Blitz S, Afilalo M, Campbell SG, Bullard M, et al. Frequency, determinants, and impact of overcrowding in emergency departments in Canada: a national survey. Healthc Q. 2007;10(4):32-40. 10.12927/hcq.2007.19312 18019897

[6] .Morley C, Unwin M, Peterson GM, Stankovich J, Kinsman L. Emergency department crowding: a systematic review of causes, consequences, and solutions. PLoS One. 2018;13(8):e0203316. 10.1371/journal.pone.0203316 PMC6117060 30161242

[7] .Dolton P, Pathania V. Can increased primary care access reduce demand for emergency care? Evidence from England’s 7-day GP opening. J Health Econ. 2016;49:193-208. 10.1016/j.jhealeco.2016.05.002 27395472

[8] .Portnoy J, Waller M, Elliott T. Telemedicine in the era of COVID-19. J Allergy Clin Immunol Pract. 2020;8(5):1489-91. 10.1016/j.jaip.2020.03.008 PMC7104202 32220575

[9] .Accorsi TA, De Amicis K, Brígido AR, Belfort DS, Habrum FC, Scarpanti FG, et al. Assessment of patients with acute respiratory symptoms during the COVID-19 pandemic by telemedicine: clinical features and impact on referral. einstein (São Paulo). 2020;18:eAO6106. 10.31744/einstein_journal/2020AO6106 PMC7690926 33295428

[10] . Birkmeyer JD, Barnato A, Birkmeyer N, Bessler R, Skinner J. The impact of the COVID-19 pandemic on hospital admissions in the United States. Health Aff (Millwood). 2020;39(11):2010-17. 10.1377/hlthaff.2020.00980 PMC7769002 32970495

[11] .Struyf T, Deeks JJ, Dinnes J, Takwoingi Y, Davenport C, Leeflang MM, Spijker R, Hooft L, Emperador D, Dittrich S, Domen J, Horn SR, Van den Bruel A; Cochrane COVID-19 Diagnostic Test Accuracy Group. Signs and symptoms to determine if a patient presenting in primary care or hospital outpatient settings has COVID-19 disease. Cochrane Database Syst Rev. 2020;7(7):CD013665. Update in: Cochrane Database Syst Rev. 2021;2:CD013665. 10.1002/14651858.CD013665 PMC7386785 32633856

[12] .Fatehi F, Martin-Khan M, Gray LC, Russell AW. Design of a randomized, non-inferiority trial to evaluate the reliability of videoconferencing for remote consultation of diabetes. BMC Med Inform Decis Mak. 2014;14:11. 10.1186/1472-6947-14-11 PMC3925960 24528569

[13] .Singal BM, Hedges JR, Radack KL. Decision rules and clinical prediction of pneumonia: evaluation of low-yield criteria. Ann Emerg Med. 1989;18(1):13-20. 10.1016/s0196-0644(89)80304-x 2642673

[14] .Hooker EA, Mallow PJ, Oglesby MM. Characteristics and trends of Emergency Department visits in the United States (2010-2014). J Emerg Med. 2019; 56(3):344-51. 10.1016/j.jemermed.2018.12.025 30704822

[15] .Fendrick AM, Saint S, Brook I, Jacobs MR, Pelton S, Sethi S. Diagnosis and treatment of upper respiratory tract infections in the primary care setting. Clin Ther. 2001;23(10):1683-706. Review. 10.1016/s0149-2918(01)80137-5 11726004

[16] .Shah SN, Bachur RG, Simel DL, Neuman MI. Does this child have pneumonia? the rational clinical examination systematic review. JAMA. 2017;318(13): 462-71. Review. 10.1001/jama.2017.9039 28763554

[17] .West JV. Acute upper airway infections. Br Med Bull. 2002;61(1):215-30. Review. 10.1093/bmb/61.1.215 PMC7110019 11997308

[18] .Malesker MA, Callahan-Lyon P, Ireland B, Irwin RS; CHEST Expert Cough Panel. Pharmacologic and nonpharmacologic treatment for acute cough associated with the common cold: chest expert panel report. Chest. 2017;152(5):1021-37. Review. 10.1016/j.chest.2017.08.009 PMC6026258 28837801

[19] .Shih J, Portnoy J. Tips for seeing patients via telemedicine. Curr Allergy Asthma Rep. 2018;18(10):50. Review. 10.1007/s11882-018-0807-5 30112587

[20] .Sociedade Brasileira de Pneumologia e Tisiologia. Diretrizes para Pneumonias Adqüiridas na Comunidade (PAC) em Adultos Imunocompetentes. J Bras Pneumol. 2004;30:S1-S24. 18833653

[21] .Parshall MB, Schwartzstein RM, Adams L, Banzett RB, Manning HL, Bourbeau J, Calverley PM, Gift AG, Harver A, Lareau SC, Mahler DA, Meek PM, O’Donnell DE; American Thoracic Society Committee on Dyspnea. An official American Thoracic Society statement: update on the mechanisms, assessment, and management of dyspnea. Am J Respir Crit Care Med. 2012;185(4):435-52. 10.1164/rccm.201111-2042ST PMC5448624 22336677

[22] .Fernández Mondéjar E. Considerations on the low adherence to clinical practice guidelines. Med Intensiva. 2017;41(5):265-6. Spanish. 10.1016/j.medin.2017.04.001 28499614

[23] .Ebell MH, Chupp H, Cai X, Bentivegna M, Kearney M. Accuracy of signs and symptoms for the diagnosis of community-acquired pneumonia: a meta-analysis. Acad Emerg Med. 2020;27(7):541-53. 10.1111/acem.13965 32329557

[24] .Pedrotti CH, Accorsi TA, De Amicis K, Serpa Neto A, Lira MT, Morbeck RA, et al. Antibiotic stewardship in direct-to-consumer telemedicine consultations lead to high adherence to best practice guidelines and a low prescription rate. Int J Infect Dis. 2021;105:130-4. 10.1016/j.ijid.2021.02.020 33578013

[25] .Monaghesh E, Hajizadeh A. The role of telehealth during COVID-19 outbreak: a systematic review based on current evidence. BMC Public Health. 2020; 20(1):1193. 10.1186/s12889-020-09301-4 PMC7395209 32738884

[26] .Ribeira R, Shen S, Callagy P, Newberry J, Strehlow M, Quinn J. Telemedicine to decrease personal protective equipment use and protect Healthcare workers. West J Emerg Med. 2020;21(6):61-2. 10.5811/westjem.2020.8.47802 PMC7673903 33052823

[27] .Budd J, Miller BS, Manning EM, Lampos V, Zhuang M, Edelstein M, et al. Digital technologies in the public-health response to COVID-19. Nat Med. 2020;26(8):1183-92. Review. 10.1038/s41591-020-1011-4 32770165

[28] .Hong Z, Li N, Li D, Li J, Li B, Xiong W, et al. Telemedicine during the COVID-19 pandemic: experiences from western China. J Med Internet Res. 2020;22(5):e19577. 10.2196/19577 PMC7212818 32349962

